# Contributions of family physicians to health care services in Nigeria

**DOI:** 10.4102/phcfm.v13i1.2943

**Published:** 2021-05-19

**Authors:** Tijani I.A. Oseni, Tawakalit O. Salam, Akinfemi J. Fatusin

**Affiliations:** 1 Department of Family Medicine, Faculty of Clinical Sciences, Ambrose Alli University, Ekpoma, Nigeria; 2 Department of Family Medicine, Irrua Specialist Teaching Hospital, Irrua, Nigeria; 3 Department of Family Medicine, University College Hospital, Ibadan, Nigeria; 4 Department of Family Medicine, Federal Medical Centre, Gusau, Nigeria

**Keywords:** family physicians, health care, general outpatient clinics, NHIS, contributions, Nigeria

## Abstract

In Nigeria, family physicians are doctors with specialised training to manage a broad range of clinical conditions and pathologies when they first present, considering the psychosocial, economic, cultural and environmental context of the individual and his or her family. In Nigeria, family physicians may be found at district hospitals but are more likely to be located at tertiary health care facilities, where their roles in medical education, research and clinical services cannot be overemphasised. Many patients present to tertiary facilities with primary-care problems, bypassing primary and secondary care. They are often seen initially by family physicians in general outpatient clinics, where 70% of all problems are managed without referral to other specialists. These physicians are also in charge of most of the National Health Insurance Scheme (NHIS) clinics nationwide. They are thus the gatekeepers to the majority of tertiary hospital services.

## Introduction

Family physicians (FPs) in Nigeria provide patient-centred, comprehensive, coordinated, integrated, longitudinal and holistic care to patients irrespective of their age, gender or disease presentation, in the context of their family, culture and environment.^1^ They provide primary care to patients in primary health care (PHC) settings, district hospitals and specialist or teaching hospitals using the principles of PHC (equity, appropriate technology and self-reliance, community participation, intersectoral collaboration and integrated services).^2^

## Family physicians in district hospitals

Although most doctors in district hospitals (private, central and general hospitals) do not have specialist training in family medicine,^3^ local governments and private hospitals across the country are increasingly seeing the need to employ FPs in these hospitals. Wherever available, FPs in these hospitals render highly specialised, cost-effective, integrated, coordinated and comprehensive care (inpatient, outpatient and surgeries) to patients irrespective of their disease presentation.^1,4^ The relevance of FPs in district hospitals can be depicted by the clinical experience of one of the authors (Box 1).

BOX 1 Patient story from a district hospital.

A middle-aged male taxi driver presented with right groin swelling of four months’ duration. He had poor libido and was unable to conduct his business because of scrotal pain upon prolonged sitting. He was constrained financially and was having marital discord. The family physician (FP) seized the opportunity of the consultation to give comprehensive care to the patient and his family. He was diagnosed with a huge right inguinoscrotal hernia, type 2 diabetes mellitus and bilateral immature cataracts. He had his blood sugar controlled and a successful surgery by the FP, who linked him to a nearby ophthalmologist. His wife, who was about 6 months pregnant, also received maternal care. The patient and his wife were satisfied with the care they received, and his quality of life improved. The FP was able to manage the medical and surgical ailments of this patient and the maternal and child health needs of his family, as well as resolving the family dysfunction, in a holistic manner.

Family medicine educators have also helped to improve the impact of family medicine in district hospitals through the introduction of a Diploma in Family Medicine programme by the National Postgraduate Medical College of Nigeria.^5^ A good number of general practitioners have obtained the Diploma in Family Medicine. This is expected to improve health care services and reduce unnecessary referrals from these hospitals to tertiary centres as well as decrease morbidity and mortality across the country.

## Family physicians in tertiary health care facilities

Tertiary hospitals (national hospital, teaching hospitals, federal medical centres and specialist hospitals) are health care facilities where different specialists render clinical services. Tertiary facilities are referral centres for most district hospitals in Nigeria, especially for complicated cases. However, in Nigeria, many patients present to tertiary hospitals directly for primary care, mainly because of a dearth of primary- and secondary-care facilities as well as human resources for health.

### General outpatient clinics

There are over 50 tertiary hospitals in the country,^6^ and these hospitals have general outpatient clinics (GOPC) staffed by FPs. These GOPCs serve as the gateway to the hospitals, as most patients coming to the hospitals pass through them. Family physicians provide first-contact comprehensive and coordinated care to these patients. They manage chronic diseases such as hypertension and diabetes and communicable diseases such as HIV, tuberculosis, neglected tropical diseases and outbreaks of emerging and re-emerging infectious diseases such as Lassa fever, yellow fever and coronavirus disease 2019 (COVID-19). Over 70% of these patients are treated solely by FPs, reducing costs as well as waiting times, as it could take up to three weeks to see other specialists from the time of referral.^4^ This also reduces the workload of other specialists, as the number of patients referred to them is significantly reduced. The way in which the FP can coordinate care within a tertiary hospital environment is illustrated in Box 2.

BOX 2 Patient story from a tertiary hospital.

The special role of a family physician (FP) in tertiary health care in Nigeria can be depicted in the care of B.O., a 65-year-old retired administrative officer, referred from a secondary-care facility to the urologist on account of benign prostatic hyperplasia and elevated prostate specific antigen, to rule out prostate cancer. There was a history of low back pain, generalised body weakness and weight loss of two years’ duration, but no urinary symptoms. After further evaluation, the urologist determined that B.O. did not have prostate cancer but referred him to the orthopaedic surgeon on account of severe lumbar spondylosis. B.O.’s symptoms persisted despite treatment, so he defaulted from the clinic.

He presented at the GOPCs about six months after trying different traditional, complementary, alternative medicines and spiritual options with no relief of symptoms. The FP evaluated B.O. and requested a full blood count, which revealed anaemia, and an erythrocytes sedimentation rate, which was markedly elevated. Further evaluation revealed a positive faecal occult blood test and metastatic bone lesions on a bone scan. He was referred to the gastroenterologist for colonoscopy, but no colorectal cancer was found. At this point B.O. continued to be seen by the FP. An abdominal computed tomography revealed a malignant retroperitoneal mass with skeletal and lung metastases. Bone biopsy, done by the haematologist, was not in keeping with lymphoid neoplasm. The general surgeon also reviewed, and a diagnosis was made of a non-operable malignant retroperitoneal mass. The FP then linked B.O. with a palliative specialist.

The FP played a major role in the coordination of care between multiple specialists in a tertiary health care facility. The FP was able to understand the patient’s and family’s perspective on the illness, including spiritual issues. The FP was trained to break bad news and understood the importance of ongoing palliative care.

### National health insurance scheme

Health expenditure in Africa is mainly out-of-pocket spending.^7^ In order to achieve universal health coverage, the National Health Insurance Scheme (NHIS) was introduced in Nigeria in 2005 in the formal sector. Most tertiary hospitals across the country have their NHIS clinics attached to family medicine clinics and run by FPs. This is because FPs manage undifferentiated illnesses irrespective of age, gender or disease entity.^1^ In addition, the wide scope of the discipline and the varied illnesses attended to make the services cost-effective to the NHIS.^1^

### Medical training and research

Family physicians in tertiary hospitals also serve as researchers and educators/trainers of both undergraduates and postgraduates in Family Medicine. Acknowledging the significant contributions of family medicine to health care services in the country, the National Universities Commission and the Medical and Dental Council of Nigeria, which are charged with regulating university education and medical practice in the country, respectively, incorporated the teaching of family medicine into the undergraduate medical curriculum.^8,9^ This measure has ensured that nearly all medical graduates from Nigeria are taught the principles of family medicine in patient management.^10^

## Other contributions

Family physicians have also strengthened the health system through their contributions to emergency, rehabilitative, geriatric and well-person care. They lead the accident and emergency units of some tertiary hospitals in the country. Their role in overseeing and coordinating the care of the elderly in the very few geriatric centres in the country cannot be overemphasised.^11^ The FP plays a key role in ensuring a multidisciplinary approach in care of the elderly, by coordinating other stakeholders involved in elder care, such as family members, nurses and other specialists. Despite these important roles, there has been a progressive decline in the numbers of FPs and trainees in Nigeria because of migration overseas.

## Conclusion

The contributions of FPs to health services in Nigeria are invaluable. There is a need to train and retain FPs at all levels of health care in the country, including tertiary health institutions, where coordination of care and postgraduate medical training in Family Medicine are essential. Furthermore, there should be greater political will to engage the services of FPs at district hospitals and in support of PHC as we work towards universal health coverage.
